# Inflammatory pathology in depression and suicide: a mechanistic distillation of clinical correlates

**DOI:** 10.3389/fimmu.2024.1479471

**Published:** 2024-12-23

**Authors:** Alessandra Costanza, Andrea Amerio, Andrea Aguglia, Luca Magnani, Alberto Parise, Khoa D. Nguyen, Isabella Berardelli, Maurizio Pompili, Mario Amore, Gianluca Serafini

**Affiliations:** ^1^ Department of Psychiatry, Faculty of Medicine, Geneva University (UNIGE), Geneva, Switzerland; ^2^ Department of Psychiatry, Faculty of Biomedical Sciences, University of Italian Switzerland (USI), Lugano, Switzerland; ^3^ Department of Psychiatry, Adult Psychiatry Service, Geneva University Hospital (HUG), Geneva, Switzerland; ^4^ Department of Neuroscience, Rehabilitation, Ophthalmology, Genetics, Maternal and Child Health, Section of Psychiatry, University of Genoa, Genoa, Italy; ^5^ IRCCS Polyclinic Hospital San Martino, Genoa, Italy; ^6^ Department of Psychiatry, San Maurizio Hospital of Bolzano, Bolzano, Italy; ^7^ Geriatric-Rehabilitation Department, University Hospital of Parma, Parma, Italy; ^8^ Chinese University of Hong Kong, Hong Kong, Hong Kong SAR, China; ^9^ Department of Microbiology and Immunology, Stanford University, Stanford, CA, United States; ^10^ Department of Neurosciences, Mental Health and Sensory Organs, Suicide Prevention Centre, Sant’Andrea Hospital, Faculty of Medicine and Psychology, Sapienza University, Rome, Italy

**Keywords:** suicide, suicidal ideation, suicidal behavior, inflammation, immune system, neuroendocrine, host-microbe deregulation, clinical subtypes

## Abstract

The association between inflammation with depression and suicide has prompted many investigations of the potential contributors to inflammatory pathology in these psychiatric illnesses. However, a distillation of diverse clinical findings into an integrated framework of the possible involvement of major physiological processes in the elicitation of pathological inflammation in depression and suicide has not yet been explored. Therefore, this review aims to provide a concise synthesis of notable clinical correlates of inflammatory pathology in subjects with various depressive and suicidal clinical subtypes into a mechanistic framework, which includes aberrant immune activation, deregulated neuroendocrine signaling, and impaired host-microbe interaction. These issues are of significant research interest as their possible interplays might be involved in the development of distinct subtypes of depression and suicide. We conclude the review with discussion of a pathway-focused therapeutic approach to address inflammatory pathology in these psychiatric illnesses within the realm of personalized care for affected patients.

## 1 Pathobiology of depression and suicide

Investigation of the complex pathobiology of depression and suicide, including both suicidal ideation (SI) and behavior (SB), is pivotal to achieve advances in diagnosis, prevention, and treatment of these psychiatric illnesses. There is a variety of neurobiological hypotheses of depression and SI/SB, often involving abnormalities from multiple areas of biology with ramified interactions (1–5). Currently, neurobiological hallmarks of depression and SI/SB are of immense research interest as no current markers of such origins are of sufficient clinical utility (6–12). Moreover, a thorough understanding of molecular and cellular pathways of these two conditions can be hindered by their complex behavioral manifestations, interaction with psychosocial, cultural, and environmental factors, diverse patient subpopulations, and the presence of somatic and psychiatric comorbidities (10, 13–17).

Recent evidence of associations between inflammatory mediators with various neurological and psychiatric illnesses, including depression and suicide, has highlighted the important role of pathological inflammation in the development of these conditions (18–26). Therefore, this review aims to categorically distill major clinical observations of the presence of inflammation in depression and SI/SB into a putative etiological framework, which involves crosstalks among immunological abnormalities, neurometabolic impairment, and dysregulated host-microbe interactions.

## 2 Inflammatory hallmarks of depression and suicide

The etiological connection between inflammation, depression, and suicide was first suggested by the increased risk of these neuropsychiatric conditions in patients with inflammatory somatic illnesses (27). Depressive symptoms and SI/SB were also observed in some patients receiving immunotherapy that elicits robust inflammatory responses, such as IFN-α (28). More importantly, the presence of focal inflammation in depression- and SI/SB-associated brain structures, such as the pre-frontal and anterior cingulate cortex (29, 30), further supported the involvement of pathological inflammation in these psychiatric illnesses. Here, we aim to synthesize notable clinical observations of diverse inflammatory manifestations in depressed and suicidal patients into specific inflammatory phenotypes of different clinical subtypes of these neuropsychiatric illnesses.

### 2.1 Depression

Major depressive disorder (MDD) is characterized by the persistent presence of depressed mood, reduced interest in pleasure-seeking activities, feelings of guilt/worthlessness, low energy, poor concentration, alterations in appetite/sleep/psychomotor functions, or suicidal thoughts. MDD has been the primary focus of studies of inflammation in patients with depressive symptoms. Notably, (epi)genetic as well as proteomic alterations of circulating and central nervous system (CNS)-specific inflammatory mediators were detected in MDD patients (**Table 1**). In this regard, polymorphisms in several innate immunity-associated inflammatory cytokines, such as CCL2, IL-1β, IL-6, and TNF-α, were correlated with more severe MDD symptoms (31). Conversely, a unique variant in IL-6R was also linked to reduced risk of depression and/or psychosis (32). In agreement with these genetic analyses, protein levels of selected innate immunity-associated inflammatory cytokines, including CCL2, TNF-α, IL-6, IL-12, and IL-18, were elevated in sera of MDD patients compared to healthy individuals (33). Importantly, early MDD-onset age was linked to a systemic pro-inflammatory state (34), driven by IL-1β and TNF-α.

**Table 1:** Inflammatory features of depressive and suicidal clinical subtypes.

Reference	Neuropsychiatric condition	Patient population	Study method/ Tissue type	Main findings
Bufalino et al. (31)	MDD	125 MDD vs 275 HC (2 studies)	Systematic review	CCL2 genetic variants were linked to MDD
Bufalino et al. (31)	MDD	576 MDD vs 706 HC (4 studies)	Systematic review	IL-1β genetic variants were linked to MDD
Bufalino et al. (31)	MDD	289 MDD vs 468 HC (2 studies)	Systematic review	IL-6 genetic variants were linked to MDD
Bufalino et al. (31)	MDD	140 MDD vs 488 HC (2 studies)	Systematic review	TNF-α genetic variants were linked to MDD
Kohler et al. (33)	MDD	3212 MDD vs 2798 HC (82 studies)	Meta-analysis/Blood	CCL2/TNF-α/IL-6/IL-12/IL-18, but not IL-17, were elevated in MDD sera
Anzolin et al. (34)	MDD	234 MDD	Proteomics/Blood	Higher serum IL-1β/TNF-α levels were linked to early MDD onset
Myint et al. (35)	MDD	40 MDD vs 80 HC	Proteomics/Blood	Increased serum IFN-γ/IL-4 ratio in MDD
Davami et al. (36)	MDD	41 MDD vs 40 HC	Proteomics/Blood	Increased serum IL-17 in MDD
Mahajan et al. (37)	MDD	23 MDD vs 23 HC	Proteomics/Brain	Increased CCL2 in hippocampal dentate gyrus of MDD
Enache et al. (38)	MDD	66 MDD vs 149 HC (3 studies)	Meta-analysis/CSF	Increased CSF IL-6 in MDD
Rush et al. (39)	Melancholic MDD	55 Melancholic MDD vs 26 HC	Proteomics/Blood	Increased serum IL-6 in melancholic MDD
Sowa-Kucma et al. (40)	Melancholic MDD	74 Melancholic vs 37 Non-melancholic MDD vs 50 HC	Proteomics/Blood	Increased serum soluble IL-6R in melancholic MDD
Yang et al. (41)	Melancholic MDD	25 Melancholic vs 17 Non-melancholic MDD	Proteomics/Blood	Increased serum IL-1β in melancholic MDD
Dionisie et al. (42)	Bipolar MDD	34 Bipolar vs 83 unipolar MDD	Proteomics/Blood	Higher neutrophil-to-lymphocyte ratio in bipolar MDD
Mao et al. (43)	Bipolar MDD	61 Bipolar vs 64 unipolar MDD	Proteomics/Blood	Decreased serum TNF-α in bipolar MDD
Brunoni et al. (44)	Bipolar MDD	59 Bipolar vs 245 unipolar MDD	Proteomics/Blood	Reduced serum IL-1β/TNF-α/IL-12 and increased serum IL-6/IL-18 in bipolar MDD
Poletti et al. (45)	Bipolar MDD	76 Bipolar MDD	Proteomics/Blood	Higher serum IL-6 was linked to poor cognition in bipolar MDD
Su et al. (46)	Bipolar MDD	10 Bipolar vs 18 unipolar MDD vs 21 HC	Proteomics/Blood	No differences in serum IL-6/TNF-α among study groups
Yoon et al. (47)	Atypical depression	35 Atypical depression vs 70 MDD	Proteomics/Blood	Increased serum IL-2, but not IL-6/TNF-α, in atypical depression
Lamers et al. (48)	Atypical depression	122 Atypical depression vs 111 MDD vs 543 HC	Proteomics/Blood	Increased serum CRP, IL-6, and TNF-α in atypical depression
Song et al. (49)	SAD	20 SAD vs 20 HC	Proteomics/Blood	Increased IL-1β/TNF-α/IFN-γ production by blood cells in SAD could be reversed by phototherapy
Leu et al. (50)	SAD	15 SAD vs 15 HC	Proteomics/Blood	Increased serum IL-6 in SAD could not be reversed by phototherapy
Corwin et al. (51)	PPD	26 HC	Proteomics/Blood	Increased serum IL-1β was linked to PPD
Leu et al. (52)	PPD	45 PPD vs 251 HC	Proteomics/Blood	Increased serum CRP and IL-6 in PPD
Nicoloro-SantaBarbara et al. (54)	PPD	16 PPD vs 17 HC	Proteomics/Blood	Increased NK-FB activation was linked to PPD 2-3 years after delivery
Sowa-Kucma et al. (40)	TRD	42 TRD vs 72 non-TRD vs 50 HC	Proteomics/Blood	Increased serum soluble IL-6R in TRD
Strawbridge et al. (56)	TRD	36 TRD vs 36 HC	Proteomics/Blood	Increased serum IL-6/IL-8/TNF-α/CRP in TRD
Mendlewicz et al. (57)	TRD	372 TRD	Genetics/Blood	COX-2 genetic variant was linked to TRD
Aguglia et al. (22)	SA	133 high-lethality vs 99 low-lethality SA	Proteomics/Blood	Increased serum CRP were linked to SA lethality
Lindqvist et al. (61)	SA	63 SA vs 47 HC	Proteomics/CSF	Increased CSF IL-6 in SA
Janelidze et al. (62)	SA	47 SA vs 17 non-suicidal MDD vs 16 HC	Proteomics/Blood	Increased serum IL-6 and TNF-α in SA
Kim et al. (63)	SA	204 SA with MDD vs 97 non-suicidal MDD	Proteomics/Blood	TNF-α genetic variant was linked to SA with MDD
Kim et al. (64)	SA	36 SA with MDD vs 33 non-suicidal MDD vs 40 HC	Proteomics/Blood	Increased serum IL-6 in non-suicidal MDD
Isung et al. (65)	SA	43 SA vs 40 HC	Proteomics/CSF	No difference in CSF IL-6 between study groups
Lindqvist et al. (66)	SA	124 SA	Proteomics/CSF	Increased CSF IL-6 was linked to SA lethality
Isung et al. (67)	SA	58 SA	Proteomics/Blood-CSF	Increased serum/CSF IL6 were linked to SA lethality
Schiavone et al. (71)	SA	26 SA vs 10 HC	Proteomics/Brain	Increased cortical IL-6 in SA
Pandey et al. (72)	SA	24 SA with MDD vs 24 HC	Proteomics/Brain	Increased IL-1β/IL-6/TNF-α and reduced IL-1RA/IL-10 in prefrontal cortex of SA
Torres-Platas et al. (74)	SA	16 SA with MDD vs 14 HC	Genetics/Brain	Increased CCL2/IBA-1 mRNA in dorsal cingulate cortex of SA
Janelidze et al. (75)	SA	137 SA vs 43 HC	Proteomics/Brain	Decreased CSF CCL2/TARC/MIP-1β in SA
Bokor et al. (77)	SA	1761 HC	Genetics/Saliva	IL-6 genetic variants were linked to increased lifetime SA risk
Knowles et al. (78)	SA	1882 HC	Genetics/Blood	IL-8 represents a female-specific genetic risk for SA, but not SI
Melhem et al. (79)	SA	38 SA vs 40 SI vs 37 HC	Genetics/Proteomics/Blood	Increased serum CRP and TNF-α mRNA in SA compared to SI
Vasupanrajit et al. (80)	SA	4043 SA/SI vs 12377 HC (59 studies)	Meta-analysis	Inflammation-related nitro-oxidative stress was strongly related to SA than SI
O'Donovan et al. (76)	SI	124 SI with MDD vs 124 HC	Proteomics/Blood	Increased inflammatory index (composite of TNF-α /IL-6/CRP/IL-10) in SI with MDD
Bokor et al. (77)	SI	1761 HC	Genetics/Saliva	IL-6 genetic variants were linked to increased current SI

CRP, C-reactive protein; CSF, cerebrospinal fluid; HC, healthy control; MDD, major depressive disorder; PPD, post-partum depression; SA, suicide attempt; SAD, seasonal affective disorder; SI, suicidal ideation; TRD, treatment-resistant depression.

Besides alterations that are closely related to innate-immune mediated inflammation, the adaptive immunity-associated inflammatory cytokine, IFN-γ, has also been implicated in the pathogenesis of depression, with increased expression of serum IFN-γ/IL-4 ratio in MDD patients (35). Another T lymphocyte-associated inflammatory cytokine, IL-17, was reportedly elevated in MDD blood samples, with some discrepancies between reports (33, 36). While the majority of MDD studies focused on peripheral inflammatory markers, neuroinflammation in MDD has also been documented. In this regard, CCL2 expression in brain parenchyma was elevated in post-mortem brain tissues of MDD patients (37). Additionally, IL-6 level in cerebrospinal fluid (CSF) were increased in MDD subjects (38).

While the aforementioned clinical findings suggest that prototypical inflammation is prominently present in MDD, some variants of this neuropsychiatric condition might exhibit distinct inflammatory profiles (**Table 1**). In this regard, melancholic MDD was associated with elevated serum IL-1β, IL-6, and soluble IL-6R levels (39–41). Furthermore, selected inflammatory alterations were uniquely linked to bipolar MDD compared to unipolar MDD. For example, increased neutrophil-to-lymphocyte ratio (NLR), an inflammatory index, has been proposed as a diagnostic parameter to distinguish bipolar from unipolar MDD (42). Other indicators to distinguish bipolar from unipolar MDD are reduced serum levels of IL-1β, TNF-α, and IL-12, and increased serum levels of IL-6 and IL-18 (43, 44). Of mechanistic importance, increased serum IL-6 was linked to poorer cognition in bipolar MDD (45). It’s worth noting that some unique inflammatory features of bipolar MDD have been disputed. For example, no significant differences in serum TNF-α and IL-6 levels were observed between bipolar and unipolar MDD (46). These differences might be related to disease activity at the time of inflammatory marker measurements. Hence, further longitudinal analyses of fluctuations in inflammatory markers of MDD patients might provide important insights on the role of specific inflammatory pathways in the onset of manic episodes.

Similar to MDD, inflammatory pathology has been observed in other depression subtypes (**Table 1**). In atypical depression, circulating inflammatory mediators, such as IL-2, C-reactive protein (CRP), IL-6, and TNF-α, were significantly elevated, although differences in IL-6 and TNF-α were not consistently observed (47, 48). In seasonal affective disorder (SAD), production of IL-1β, TNF-α, and IFN-γ in cultures of circulating immune cells was elevated, which could be reversed by phototherapy (49). Interestingly, serum IL-6 was also elevated in SAD patients but could not be normalized by this therapeutic approach (50), suggesting distinct effects of this treatment on different inflammatory mediators. In post-partum depression (PPD), depressive symptoms were associated with increased expression of serum CRP, IL-1β, and IL-6 (51, 52). Notably, elevated inflammation was detected as early as 1-3 days after childbirth (53) and their persistence for several years after delivery was linked to PPD (54), highlighting the possible initiating role of inflammation in this type of depression as well as its long-lasting epigenetic impact.

Treatment responsiveness represents a therapeutic challenge for patients with depression (55), partly owing to the delayed impact of anti-depressants that occur only after an extended treatment period (of at least one month). Consequentially, pre-treatment biomarkers that could predict therapeutic efficacy of anti-depressants are of immense clinical interest. In this regard, several predictors of treatment-resistant depression (TRD) have been proposed, including elevated serum levels of IL-6, IL-8, TNF-α, soluble IL-6R, and CRP (40, 56) or polymorphisms in COX-2 (57). However, some conflicting findings regarding correlations between changes in inflammatory markers and antidepressant response to ketamine in TRD exist (58), which might be explained by the heterogeneity of this patient population. In fact, anti-inflammatory therapy was only effective in a subset of TRD patients with elevated expression of inflammatory mediators (59).

### 2.2 Suicidal ideation and behavior

The interconnected pathobiology of depression and suicide suggests that overlapping inflammatory signatures might exist between these two conditions. However, few studies have examined potential differences in inflammatory features between suicidal depressed and suicidal non-depressed subjects (24). It has been hypothesized that subjects at risk for suicide might be biologically distinguishable from depressed individuals without such risk by a heightened pro-inflammatory state, characterized by elevated serum IL-6 (60). Nevertheless, a consensus on unique and/or shared inflammatory features between depression and SI/SB has not been reached, given the diverse approaches to patient stratification in studies of these two conditions. As such, mechanistic insights from clinical correlates must be carefully interpreted. The following section aims to highlight inflammatory changes in different suicidal subtypes, while acknowledging the possible coexistence of depressive symptoms in affected individuals.

In subjects who made an suicidal attempt (SA), increased serum levels of IL-6 and TNF-α, elevated CSF levels of IL-6, and TNF-α polymorphisms were observed (61–63). However, differences in IL-6 in CSF and sera could not be confirmed by other studies (64, 65), possibly due to confounding demographic factors, such as patient stratification and/or medication status. Interestingly, the lethality of SA was reportedly associated with specific inflammatory chemokines/cytokines in the CSF (66) as well as serum CRP and IL-6 (a stimulator of hepatic CRP synthesis) (22, 67). Mechanistically, increased expression of inflammatory markers might predispose subjects with specific behavioral traits, such as impulsivity, aggression, and hostility (15, 68–70), to violent SA.

Besides fluid-based biomarker studies, post-mortem brain tissue analysis has provided important insights on the possible association between neuroinflammation and SA. For example, cortical IL-6 was upregulated in asphytic suicidal subjects (71). Depressed suicidal patients also showed elevated expression of inflammatory cytokines (IL-1β, IL-6, and TNF-α) and reduced levels of anti-inflammatory mediators (IL-1RA and IL-10) in the prefrontal cortex (72). Interestingly, aberrant epigenetic regulation might account for the elevated expression of TNF-α in the brains of subjects with SA (73). Increased CCL2 mRNA expression were also noted in the dorsal anterior cingulate cortex of suicide victims, which was correlated with IBA-1-expressing brain macrophage accumulation (74). However, decreased macrophage-attracting chemokines, including CCL2, TARC, and MIP-1β, were observed in another study (75).

In MDD subjects with SI, a higher systemic inflammatory score was observed compared to healthy controls (76). Furthermore, IL-6 genetic variants appeared to interact with childhood adversities or recent life stress to influence lifetime risk of SA and current SI (77). However, different magnitudes of inflammatory changes might distinguish SA from SI. For example, the chemokine IL-8 represented a female-specific genetic risk factor for SA but not SI (78). Increased serum CRP and TNF-α mRNA from blood cells were also observed in SA compared to SI (79). Finally, inflammation-related nitro-oxidative stress was more strongly related to SA than SI (80).

While these observations need to be further validated with detailed consideration of patient characteristics and other confounders, they have prompted a provocative hypothesis that dynamic changes in inflammation might underlie the development of SI/SA and progression from the former to the latter. Furthermore, inflammatory pathology has also been documented in various psychiatric illnesses, suggesting the possible existence of a common inflammatory pathway among behavioral disorders.

## 3 Contributors to inflammation in depression and suicide

**Figure 1:**
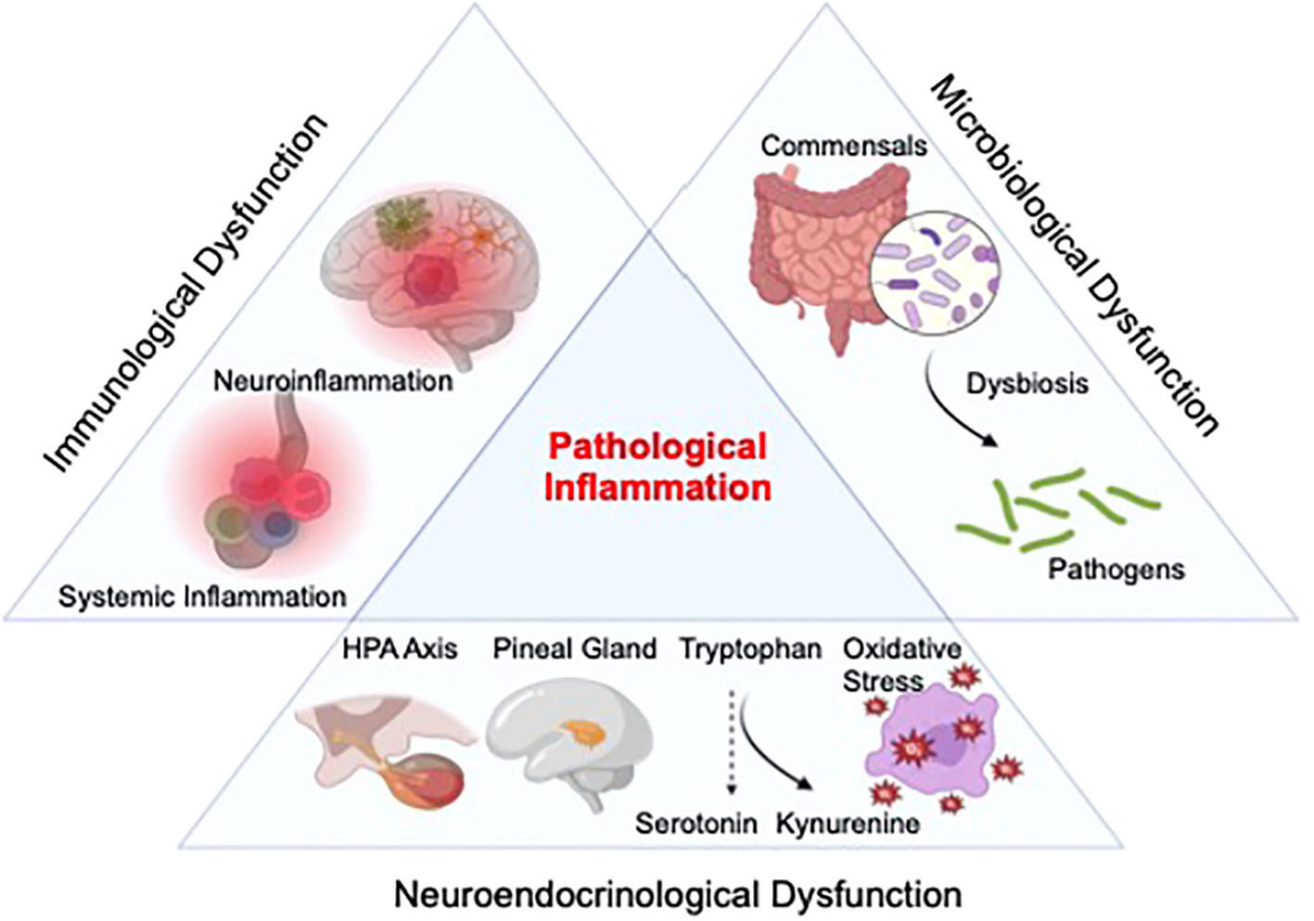
Proposed etiological framework of inflammation in depression and suicide. Inflammation in depression and suicide might result from overactivation of the neuroimmune system, with abnormalities in the function/distribution of various circulating adaptive and innate immune cell as well as possible alterations in activation profiles and concentration of cellular components of the central nervous system (CNS), including astrocytes, microglia, and infiltrated macrophages. Furthermore, alterations in several neurometabolic axes, such as the hypothalamic-pituitary-adrenal (HPA) pathway, melatonin signaling, kynurenine metabolism, and oxidative stress, might also contribute to this pathology. Lastly, dysregulated host-microbe interaction (dysbiosis) may heighten the production of inflammatory mediators in these neuropsychiatric conditions.

While the relative contribution of each inflammatory mediator to distinct pathological manifestations of depression and SI/SB needs to be further examined, abnormalities in major inflammation-associated physiological processes have been observed in these conditions. Notably, immunological and neuroendocrinological abnormalities have been linked to inflammatory pathology in depression and suicide. Furthermore, dysregulated host-microbe interaction has recently emerged as another major contributor to inflammation in these conditions. While we do not exclude the possible crosstalks between these signaling axes with other factors, such as those of (epi)genetic and environmental origins, the following sections will propose a mechanistic synthesis of clinical evidence focusing on abnormalities in these three major physiological processes (**Figure 1**).

### 3.1 Aberrant activation of the immune system

As a major source of inflammatory mediators, the immune system has long been implicated in the development of inflammatory pathology in depression and suicide (23, 24, 81). Peripheral perturbations in various immune cell subsets have been observed in these conditions. For example, reduced function and numbers of T cells and natural killer cells were linked to MDD (82). However, expansions of various T-helper subtypes, including Th-17 cells, were also detected in depressed patients (83), suggesting that temporally distinct immune activation profiles might exist during the progression of this psychiatric illness. Regarding innate immunity-related changes, increased numbers of blood monocytes and frequencies of neutrophils were observed in depressed subjects (60, 84). Notably, a heightened systemic accumulation of these cells might distinguish subjects with increased suicide risk from non-suicidal depressed patients (60).

Besides alterations in peripheral immunity, abnormal activation of microglia and macrophages in the CNS has also been examined in the context of depression and suicide. However, observations of gliosis-related changes were inconsistent in depressed and suicidal patients (38), possibly due to the impact of death on glial cell reactivity as well as the different types of tissue-sampling methods (brain regions, cerebrospinal fluid *vs.* brain parenchyma). Alternatively, gliosis might have resulted from an influx of peripheral monocytes/macrophages. The latter viewpoint is supported by observations of higher concentrations of blood vessels surrounded by macrophages in the anterior cingulate cortex of depressed suicidal patients, evidenced by increased IBA-1 and CD45 mRNA expression (74). Increased activation markers of astrocytes have also been implicated in suicide and depression. For instance, increased circulating levels of S100B and CSF levels of glial fibrillary acidic protein (GFAP) were observed in depressed subjects (85, 86), while reactive astrocytosis with hypertrophic morphology was noted in the white matter of the anterior cingulate cortex of depressed suicide completers (87).

**Figure 2:**
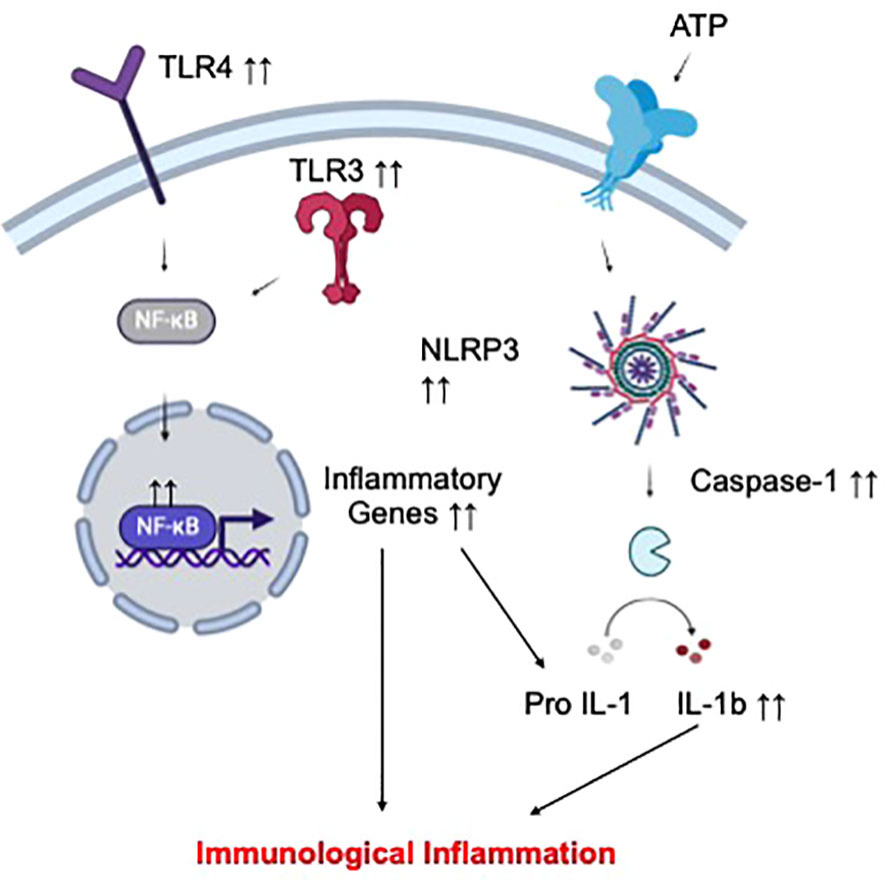
Proposed mechanism of immunological inflammation in depression and suicide. Increased activation status of different circulating and CNS immune cell subsets has been implicated in depression and suicide. Such hyperinflammatory phenotype of these cells might result from elevated signal transduction via selected pro-inflammatory pathways, including those of TLR-NFKB and the NLRP3 inflammasome, leading to overproduction of inflammatory cytokines, and ultimately, peripheral and CNS inflammation.

Altogether, the clinical findings above highlight hyper-activation of the peripheral and CNS immune repertoires as a root cause of pathological inflammation in depression and suicide. Mechanistically, exuberant inflammatory responses of peripheral and CNS immune cells in the context of these psychiatric conditions might be mediated by abnormally activated pro-inflammatory signaling cascades (**Figure 2**). In this regard, increased expression of various molecular regulators of the toll-like receptors (TLR)/nuclear factor-kappa B (NF-KB) pathway have been associated with depression and suicide. For example, increased TLR4 expression in peripheral immune cells was linked to MDD symptoms while elevated TLR3/TLR4 expression was observed in the dorsolateral prefrontal cortex of depressed suicidal patients (88, 89). NF-KB and the expression of its related genes were reportedly higher in depressed adolescents and adults (90, 91). Another master regulator of inflammation, the nod-like receptor pyrin-containing 3 (NLRP3) inflammasome, has also been implicated in these neuropsychiatric conditions (92). For example, mRNA expression of NLRP3 and its related genes (the adaptor apoptosis-associated speck-like protein ASC, caspase-1) in peripheral immune cells were increased in MDD patients compared to healthy controls (93, 94). Specific DNA methylation pattern of NLRP3 in depressed brains was also linked to depression-associated neuroanatomical changes (cortical thickness) and neuroinflammatory processes (95). In an analysis of post-mortem brain samples from depressed suicidal patients, evidence of hyperactive NLRP3 inflammasome (elevated protein and mRNA expression of NLRP3, Caspase-1, and the inflammasome adaptor protein ASC) was also observed (96). Importantly, two anti-depressants, amitriptyline (97) and ketamine (98), could respectively reduce NLRP3 and NK-FB expressive, and consequentially, inflammatory cytokine production in peripheral blood cells from MDD subjects. Collectively, these findings provide further mechanistic support for the involvement of pro-inflammatory signaling cascades in the orchestration of immunological inflammation in MDD.

### 3.2 Impaired neurometabolic signaling

**Figure 3:**
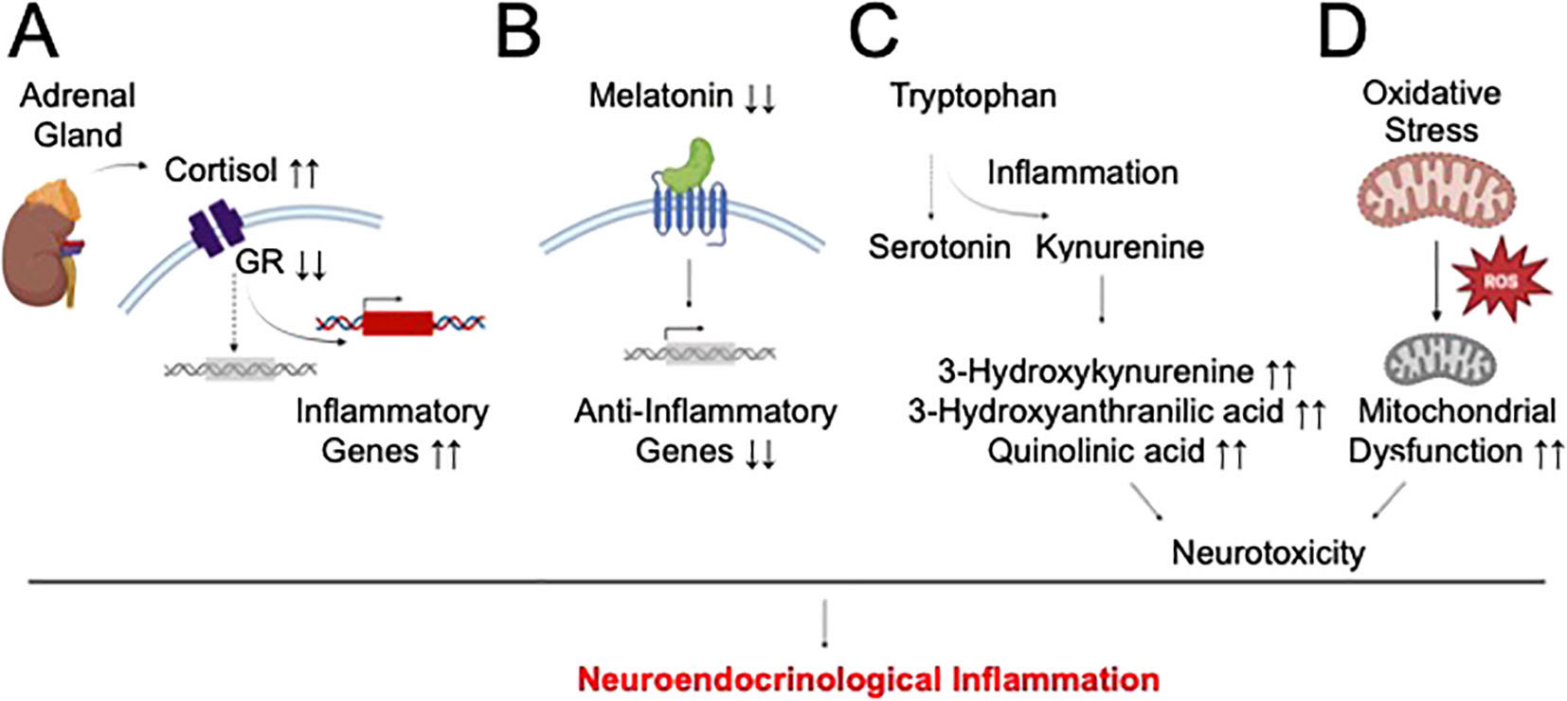
Proposed mechanism of neuroendocrinological inflammation in depression and suicide. Several neuroendocrine pathways might contribute to the inflammatory pathology of depression and suicide by distinct pathways. **(A)** HPA dysfunction-induced hypercortisolemia might result in glucocorticoid receptor (GR) downregulation/desensitization and activation of non-canonical inflammation signaling of cortisol. **(B)** Impaired melatonin production might dampen its downstream anti-inflammatory properties. **(C)** Primary inflammatory insult might induce the divergence of tryptophan’s catabolic fate away from serotonin synthesis and towards neurotoxic kynurenine metabolism, and ultimately, secondary inflammation. **(D)** Oxidative stress might result in mitochondrial dysfunction and impaired antioxidant defense, causing neurotoxicity and inflammation.

Quantitative analyses of clinical specimens from depressed and suicidal subjects revealed changes in the concentrations of various metabolites and hormones compared to healthy individuals. Importantly, these bioactive molecules point to the potential disruption of homeostatic signaling of several neuroendocrine systems as another important contributor to pathological inflammation in these neuropsychiatric illnesses (**Figure 3**). Their putative mechanistic contributions to pathological inflammation in depression and suicide will be detailed in this section.

Abnormalities in the hypothalamic-pituitary-adrenal (HPA) axis, a major stress response system, have been noted in depression and suicide, regardless of the presence of any other psychiatric conditions (99). This signaling pathway consists of various neuroendocrine hormones, such as corticotropin-releasing hormone (CRH), adrenocorticotropic hormone (ACTH), and cortisol, which are released in response to stress. In this regard, more than 40% of depressed patients exhibited hypercortisolemia, increased CRH production or reduced ACTH release (100), with hypercortisolemia being associated with MDD severity and MDD-associated psychosis (101). Similarly, increased cortisol response has been observed in depressed suicidal subjects (102). Age-dependent hypercortisolemia were also linked to SA (103). Mechanistically, hypercortisolemia might represent a compensatory response to reduced glucocorticoid receptor (GR) expression. In fact, reduced hippocampal GR expression was observed in brain specimens of abused suicidal patients and linked to early-life trauma-induced epigenetic changes (methylation) in the NR3C1 gene (104). Alternatively, hypercortisolemia might desensitize GR signaling (105), thereby increasing the propensity for non-canonical inflammatory signaling of cortisol (**Figure 3A**). In fact, reduced GR-α expression in the prefrontal cortex and amygdala of suicide victims was linked to higher expression of CRP and TNF-α (106).

Beside HPA dysfunction, perturbations in the signaling pathway of melatonin, a pineal gland-derived hormone that regulates the sleep-wakefulness cycle, have been observed in depression and suicide. Specifically, single-nucleotide polymorphism of the melatonin receptor in the brain was associated with depression (107), while changes in melatonin levels and production patterns was documented in various depression subtypes (108). Decreased melatonin concentrations in the pineal gland was also observed in suicide victims (109). A connection between melatonin dysregulation and inflammatory pathology in depression and suicide might be related to the compromised anti-inflammatory properties of this hormone on innate immune cells (110) (**Figure 3B**).

Another prominent hypothesis of depression and suicide development focuses on dysregulated kynurenine metabolism. This metabolite of the tryptophan catabolic pathway represents a rate-limiting step in the diversion of tryptophan availability from the synthesis of serotonin, a neuroprotective neurotransmitter in depression. In fact, acute tryptophan deprivation might be able to trigger clinical symptoms of depression (111). Additionally, kynurenine pathway activation was observed in various forms of depression (112, 113) and correlated with depression severity (114). Increased toxic metabolites of the kynurenine pathway, such as 3-hydroxykynurenine, 3-hydroxyanthranilic acid, and quinolinic acid, have been associated with reduced cortical thickness in MDD (115) or linked to MDD development (116, 117). In suicidal patients, reduced serotonin metabolism was linked to possible dysregulation of kynurenine signaling (118). In fact, positive correlations between the cytokine activation marker neopterin with the kynurenine:tryptophan ratio and between abnormally elevated quinolinic acid concentration and increased inflammatory potential have been reported (119, 120). Elevated serum concentration of kynurenine was also observed in MDD patients with SA (121). Of mechanistic interest, a bidirectional interaction between kynurenine metabolism and inflammation might occur in depression and suicide. While kynurenine catabolic activation might be induced by inflammatory signals, toxic metabolites of this pathway might cause neuronal injuries and secondary inflammatory response, thereby propagating this vicious interactome (**Figure 3C**).

Finally, oxidative stress has been implicated in depression and suicide (23, 24, 122). Reduced antioxidant levels and/or increased oxidative stress markers were observed in brain specimens and blood samples of depressed subjects (122, 123). In suicide pathophysiology, increased NADPH oxidase was associated with SI, while increased nitro-oxidative stress markers and reduced antioxidant levels were linked to SA (80, 124). Mechanistically, oxidative stress can damage the mitochondria, triggering inflammatory reactions (**Figure 3D**). In fact, concomitant increases in inflammation and oxidative stress were observed in maltreated children, who developed higher risk of depression in adulthood (125). Reduced circulating antioxidants in depressed patients were also linked to elevated serum inflammatory cytokines (126). Furthermore, the high-oxygen demand of the CNS renders this tissue highly susceptible to oxidative stress-induced mitochondrial damage and reduced antioxidant defense, followed by the vicious cycle of elevated neuroinflammation and neurotoxicity in vulnerable brain regions, which underlies various behavioral pathologies of depression and suicide development.

### 3.3 Dysregulated host-microbe interaction

Alterations in the microbiome have recently emerged as a novel pathology of various neuropsychiatric illnesses, including depression and suicide (127, 128). In depressed subjects, the most commonly observed alterations in the microbiome are related to those of the gastrointestinal compartment. In this regard, most studies found changes in beta-diversity (dissimilarity between depressed and control subjects) as well as alterations in abundance (the concentrations of specific microbes) of selected microbial phyla (*Proteobacteria* and *Fusobacteria*), families (*Rikenellaceaea*, *Enterobacteriaceae*, and *Ruminococcaeceae*), and geniuses (*Eggerthella, Alistipes, Parabacteroides, Enterococcus*, *Lactobacillus, Veillonella, Flavonifractor*, *Streptococcus* and *Faecalibacterium*) in fecal samples of MDD patients compared to health controls (129). In other studies, alterations in abundance, but not diversity, of selected salivary microbial genera (*Prevotella, Haemophilus, Rothia, Treponema, Schaalia, Neisseria, Solobacterium, Lepotrichia, Fusobacterium*, and *Veillonella*) and species (*Lachnoanaerobaculum orale, Fusobacterium periodonticum*, and *Mobiluncus Mulieris*) were associated with depressive symptoms (130, 131). In suicidal MDD patients, six microbial taxa were linked to suicidal behaviors (positive associations: *Hungatella* and *Fusicatenibacter*, negative associations: *Butyricicoccus, Clostridium, Parabacteroides merdae*, and *Desulfovibrio piger*) (132). Furthermore, SI was reportedly associated with changes in salivary abundance of various microbial genera (*Veillonella, Streptococcus, Prevotella, Rothia, Alloprevotella, Granulicatella, Porphyromonas, Peptostreptococcus, Stomatobaculum, Gemella*, and *Solobacterium*) and species (*Streptococcus oralis, Prevotella melaninogenica, Fusobacterium periodoncitum, Rothia mucilagniosa, Graulicatella adiacens, Streptococcus parasanguinis, Prevotella nanciensis, Solobacterium moorei, Dialister pneumosintes, Peptoanaerobacter stomatis, Actinomyces naeslundii, Bacteroides dorei*, and *Fusobacterium naviforme)* (133). Last but not least, increased laxative misuse, which is associated with gastrointestinal microbial dysbiosis, were linked to a history of SA (134).

**Figure 4:**
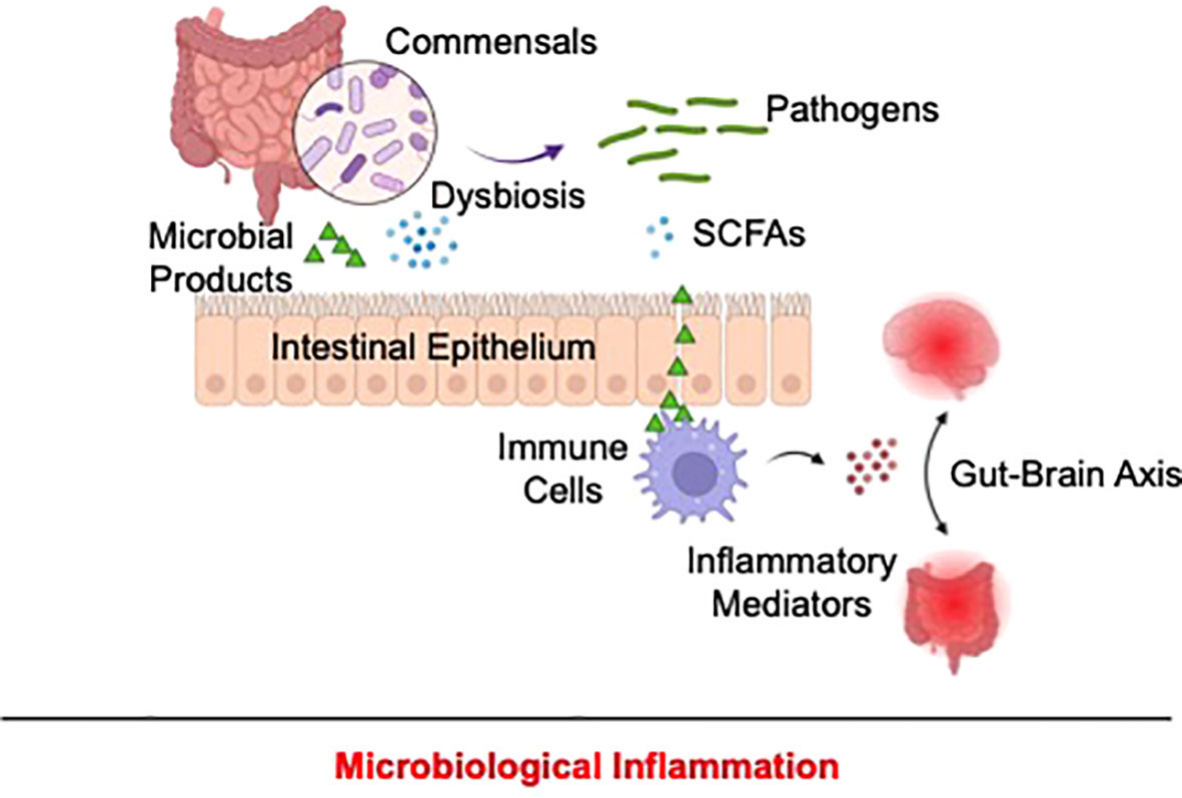
Proposed mechanism of microbiological inflammation in depression and suicide. In depression and suicide, alterations of the microbiome might disrupt intestinal barrier, allowing exposure of resident immune cells to pathogen-associated pro-inflammatory stimuli and concomitantly reducing the production of anti-inflammatory microbial products, including as short-chain fatty acids. As a result, unrestrained gastrointestinal immune activation occurs and might be relayed to the CNS via the gut-brain interactome, resulting in depression and suicide-associated inflammation.

Mechanistically, microbial dysbiosis might contribute to inflammatory pathology by triggering the loss of intestinal barrier integrity, exposing intestinal immune cells to inflammatory environmental stimuli. In fact, higher depressive symptoms were reportedly associated with increased intestinal permeability in cancer patients (135). Reduced zonulin expression, a biomarker of increased intestinal permeability, was also observed in patients with MDD or those with SA (136). Furthermore, compositional changes in commensals, as a result of dysbiosis, might trigger excessive release of microbe-associated inflammatory stimuli (137) and reduce the availability of anti-inflammatory microbial products, such as short-chain fatty acids (138), leading to unrestrained intestinal immune activation. Of note, the gut-brain interactome might relay microbiome-associated inflammatory signals from peripheral tissues to the brain (137, 139), contributing to depression- and suicide-associated neuroinflammation (**Figure 4**).

## 4 Therapeutic insights

Current literature on the involvement of inflammation and various putative etiological pathways of this pathology in depression and suicide has prompted clinical examination of the efficacy of anti-inflammatory agents for the treatment of these psychiatric conditions. Early trials primarily focused on non-steroidal anti-inflammatory drugs (NSAIDs). NSAIDs’ primary mechanism of action is to inhibit the enzyme cyclooxygenase (COX), which is responsible for the production of inflammatory eicosanoids (thromboxanes, prostaglandins, and prostacyclins). As a standalone (140, 141) or adjunct therapy (142, 143), NSAIDs yielded promising outcomes as a treatment for depressive symptoms in MDD patients as well as those with other comorbid illnesses. NSAIDs were also suggested to be effective in mitigating SI risk (26), providing the impetus for further exploration of these agents in suicide prevention. Besides NSAIDs, glucocorticoids, whose binding on their cognate receptor, GR, turns on anti-inflammatory gene expression to curb immune cell activation, also exhibited short-term efficacy in alleviating depression (144, 145). Minocycline, an antibiotic with dual immunosuppressive function via its inhibition of the inflammasome (Caspase-1 and Caspase-3), pro-inflammatory enzymes (COX-2, inducible nitric oxide synthase [iNOS], matrix metalloproteinase [MMP], and phospholipase A2 [PLA_2_]), and immune cell proliferation/activation, also exerted anti-depressant effects in TRD patients and depressed HIV-infected subjects (146, 147). However, the efficacy of these agents requires further clinical validation (148). To date, the most compelling evidence for a pathway-focused treatment of depression, with mechanistic relevance to inflammatory pathology, emerged in clinical studies of inhibitors against different inflammatory cytokines (148). For example, IL-6 inhibitors (sirukumab and siltuximab) (149), IL-12/IL-23 inhibitor (ustekinumab) (150), IL-17 inhibitor (ixekizumab) (151), and TNF-α inhibitors (adalilumab and etanercept) (152–155) were effective in reducing depressive symptoms in patients with comorbid inflammatory diseases. Notably, infliximab, another TNF-α inhibitor, also showed some promising anti-depressant effect in TRD patients (60).

Besides these immune-related therapies, microbiome modifications have emerged as an attractive therapeutic promise in depression. Some proprietary probiotic formulations (live microorganisms with health-promoting benefits) were reportedly effective in reducing depressive symptoms (156–159). Additionally, prebiotics (nutrients that promote the growth of beneficial microorganisms) might possess some anti-depressant effects, with conflicting outcomes that require further confirmation (159, 160). Various molecular regulators of oxidative stress, HPA axis, and kynurenine pathway have also been proposed as promising therapeutic targets in depression treatment and suicide prevention (126, 161). Additionally, the impact of melatonin on depression-related inflammation warrants further examination, given its preliminary clinical effectiveness in depression (162). Other treatment approaches for depression focused on anti-infective agents (163) and their potential inhibitory effect on inflammation. Notably, melatonin and some anti-infective/anti-inflammatory therapies have been linked to the development of depressive symptoms and SB/SI, prompting additional caution when using these agents to treat psychiatric illnesses (164–166). Lastly, other treatment approaches with anti-inflammatory properties, such as psilocybin and cognitive behavioral therapies (167, 168), might provide additional mechanistic insights on the origin of inflammation in depression and suicide.

Given the diverse nature of inflammatory phenotypes of different subtype of depression and suicide, future treatment approaches might be more clinically impactful with detailed considerations of unique alterations in inflammatory biomarkers in prescribing anti-inflammatory treatments. Such biomarkers might also be of utility in monitoring treatment responsiveness, which are of urgent need in the case of TRD. Furthermore, the co-existence of dysfunctions in multiple inflammatory signaling pathways might require a combination treatment approach to achieve optimal therapeutic outcomes. Finally, integrated care that combines interventions addressing specific inflammatory pathology with current psychopharmacologic and psychotherapeutic treatments, is expected to improve therapeutic efficacy in depression and suicide.

## 5 Conclusions

The development of inflammatory pathology in depression and suicide may stem from dysregulation in immunological and neuroendocrinological signaling and improper microbial interactions with the host. Further investigations are required to elucidate the precise contributions of and/or interactions among these major physiological processes in distinct clinical subtypes of depression and suicide. These questions are of great clinical interest as they could help identify disease patterns, in the context of pathological inflammation, that may allow more targeted treatment approaches and personalized preventive strategies for depression and suicide.
